# The Regulation of miRNA-211 Expression and Its Role in Melanoma Cell Invasiveness

**DOI:** 10.1371/journal.pone.0013779

**Published:** 2010-11-01

**Authors:** Joseph Mazar, Katherine DeYoung, Divya Khaitan, Edward Meister, Alvin Almodovar, James Goydos, Animesh Ray, Ranjan J. Perera

**Affiliations:** 1 Sanford Burnham Medical Research Institute, Orlando, Florida, United States of America; 2 Curtis and Elizabeth Anderson Cancer Institute, Savannah, Georgia, United States of America; 3 Robert Wood Johnson Medical School, Cancer Institute of New Jersey, New Brunswick, New Jersey, United States of America; 4 Keck Graduate Institute, Claremont, California, United States of America; Cleveland Clinic, United States of America

## Abstract

The immediate molecular mechanisms behind invasive melanoma are poorly understood. Recent studies implicate microRNAs (miRNAs) as important agents in melanoma and other cancers. To investigate the role of miRNAs in melanoma, we subjected human melanoma cell lines to miRNA expression profiling, and report a range of variations in several miRNAs. Specifically, compared with expression levels in melanocytes, levels of miR-211 were consistently reduced in all eight non-pigmented melanoma cell lines we examined; they were also reduced in 21 out of 30 distinct melanoma samples from patients, classified as primary *in situ*, regional metastatic, distant metastatic, and nodal metastatic. The levels of several predicted target mRNAs of miR-211 were reduced in melanoma cell lines that ectopically expressed miR-211. *In vivo* target cleavage assays confirmed one such target mRNA encoded by *KCNMA1*. Mutating the miR-211 binding site seed sequences at the KCNMA1 3′-UTR abolished target cleavage. KCNMA1 mRNA and protein expression levels varied inversely with miR-211 levels. Two different melanoma cell lines ectopically expressing miR-211 exhibited significant growth inhibition and reduced invasiveness compared with the respective parental melanoma cell lines. An shRNA against KCNMA1 mRNA also demonstrated similar effects on melanoma cells. miR-211 is encoded within the sixth intron of *TRPM1*, a candidate suppressor of melanoma metastasis. The transcription factor MITF, important for melanocyte development and function, is needed for high *TRPM1* expression. MITF is also needed for *miR-211* expression, suggesting that the tumor-suppressor activities of MITF and/or *TRPM1* may at least partially be due to miR-211's negative post transcriptional effects on the KCNMA1 transcript. Given previous reports of high KCNMA1 levels in metastasizing melanoma, prostate cancer and glioma, our findings that miR-211 is a direct posttranscriptional regulator of KCNMA1 expression as well as the dependence of this miRNA's expression on MITF activity, establishes miR-211 as an important regulatory agent in human melanoma.

## Introduction

Melanoma, a cancer of the pigment-producing cells in the skin epidermis, can be highly metastatic, and malignant melanomas are relatively resistant to standard chemotherapy [1]. A major cause for melanoma initiation is extensive or intermittent exposure to the sun's radiation over a period of time, and the extent of melanin pigmentation is an important risk factor [2]. The exact molecular mechanisms that lead to melanoma are complex and poorly understood [3]–[6], and may involve both mutagenic DNA lesions and epigenetic misregulation [7]–[11]. The complexity is added by the involvement of several different signal transduction pathways, such as the *Hedgehog* pathway, which controls BCL2-mediated apoptosis; mutations in the *Patched* gene, the endpoint of the *Hedgehog* pathway, have been correlated with skin cancers [3], [12]–[15]. A frequent causative mechanism for an inherited form of predisposition to melanoma is thought to be a chromosomal deletion over 9p21. The 9p21 site harbors the tumor suppressor gene INK4a and accompanies additional inactivating mutations that lead to the constitutive activation of genes such as *BRAF* [16], [17]. INK4a encodes one of several cyclin-dependent protein kinase inhibitors, which is located adjacent to an alternate reading frame of the human p14^ARF^. P14^ARF^ binds to the Mdm2 protein in several cell lines (though remains untested in melanoma cell lines, to our knowledge) and thereby abrogates Mdm2's binding to p53, causing p53 to be stabilized and nuclear localized. The loss of INK4a therefore may lead to interference of two separate pathways of cell cycle control: CDK signaling and suppression of p53 activity by Mdm2-induced acceleration of p53 degradation. Methylation near the 5′ upstream region of INK4a has been shown in some 10% of melanomas [7], suggesting that epigenetic down-regulation of this gene may be important for melanoma development. The activation of *BRAF* alone may be insufficient to cause metastatic melanoma, but additional mutagenic or epigenetic events such as the inactivation of tumor suppressor genes, *e.g.*, Pten [18], may be important. There is evidence that the *NOTCH* signalling pathway is important for distinguishing normal melanocytes from melanoma cells [19], [20]. Measurement of genome-wide DNA copy number variations, together with analysis of somatic mutations in specific marker genes, can be used to distinguish among different melanoma subtypes with reasonable accuracy [21]. Particularly noteworthy is the recent demonstration of abnormally high oncogenic potentials of single melanoma cells [22], emphasizing the need for better understanding the molecular mechanisms of melanoma progression.

Attention has recently focused on the role of small non-coding RNA molecules in cancer development [23]–[27] and in melanoma in particular [28]–[32]. miRNAs influence cancer development by serving either as tumor suppressors or oncogenes [33]–[39] by their negative regulatory effects on mRNA encoded by oncogenes or tumor suppressor genes, respectively. With the goal of defining the genes with major contributions to melanoma, several genome-wide expression level studies have identified a number of protein-coding [40] and microRNA (miRNA) genes as important players [32], [41]–[43]. Several of these genes and their expression signatures exhibit distinct patterns among malignant metastatic melanomas and their benign forms, but their significance with respect to melanoma initiation and progression is poorly understood. For example, miR-221/222 were found to down-regulate p27Kip1/CDKN1B and the c-KIT receptor, which controls the progression of neoplasia leading to enhanced proliferation and reduced differentiation in melanoma cells [42]. Similarly, high miR-137 expression in melanoma cell lines down-regulates microphthalma associated transcription factor (MITF), a transcription factor important for melanocyte cell growth, maturation, apoptosis, and pigmentation [32]. The depletion of miR-182 reduces invasiveness and induces melanoma cell death by suppressing the expression of transcription factors FOXO3 and MITF [43], suggesting that its increased expression may be associated with certain aspects of melanoma biology. Relatively less is known about the downstream genes that are regulated by MITF and FOXO3, which are evidently important for melanoma progression and metastasis.

In contrast to miRNAs that are over-expressed in melanoma, and their respective target genes that are thus under-expressed, relatively little is known about miRNA species that are systematically depleted in melanomas. Consequently, their respective target genes, expected to be up-regulated, which might explain some of the oncogenic potentials of invasive melanomas, are largely unrecognized. Realizing this gap in knowledge, we examined the expression levels of human miRNAs in defined melanoma cell lines and clinical melanoma samples. We report here the reduced expression of miR-211 in these cell lines and clinical isolates of human melanomas, and present evidence that a principal effect of the reduced expression of miR-211 is the increased expression of its target transcript KCNMA1. The expression of *KCNMA1*, encoding a calcium ion-regulated potassium channel protein, appears to at least partially account for the high cell proliferation rate and invasiveness of melanoma cell lines. We also demonstrate that MITF expression is important for the coordinate expression of *miR-211*, and *TRPM1*. *TRPM1* gene is a suppressor of melanoma metastasis, which encodes a transient receptor potential family member calcium channel protein, and encodes *miR-211* gene in its sixth intron. Here, we propose a model for the role of miR-211 and its regulation in melanoma cells.

## Results

### miR-211 is expressed at a low level in non-pigmented melanoma cell lines

**Figure 1:**
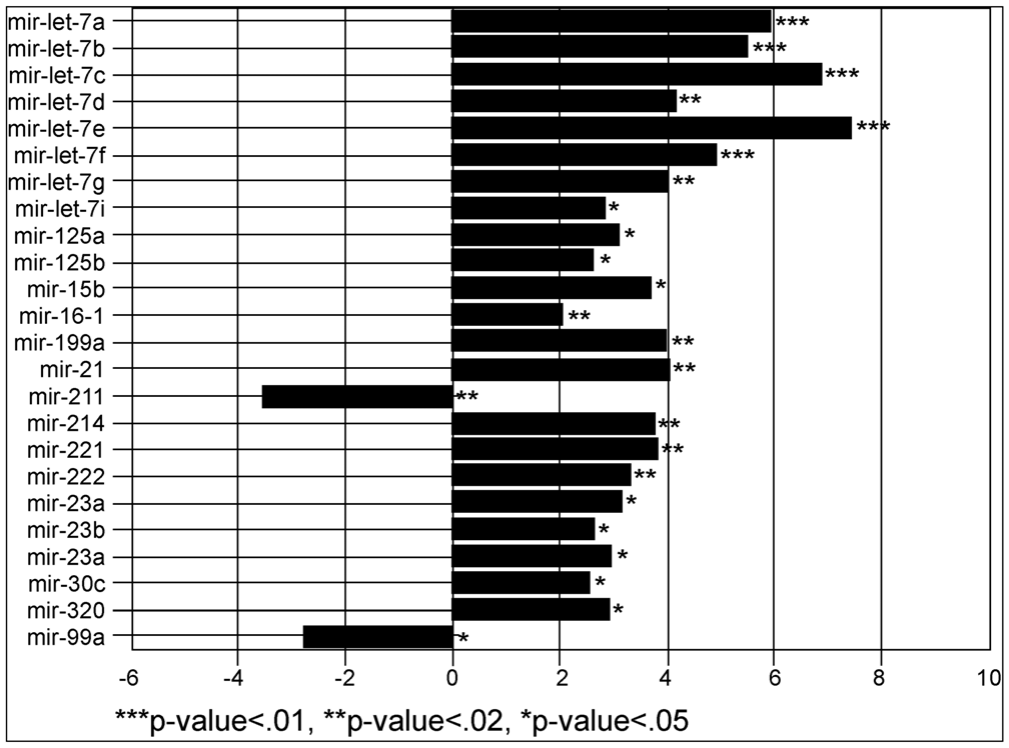
Differentially expressed miRNA transcripts in the melanoma cell line WM1552C. Histograms of log_2_ of mean expression ratios of miRNA levels in WM1552C to that in the untransformed melanocyte cell line HEM-l (control) are plotted as histograms. Asterisks indicate the respective levels of statistical significance, indicated below the diagram.

**Figure 2:**
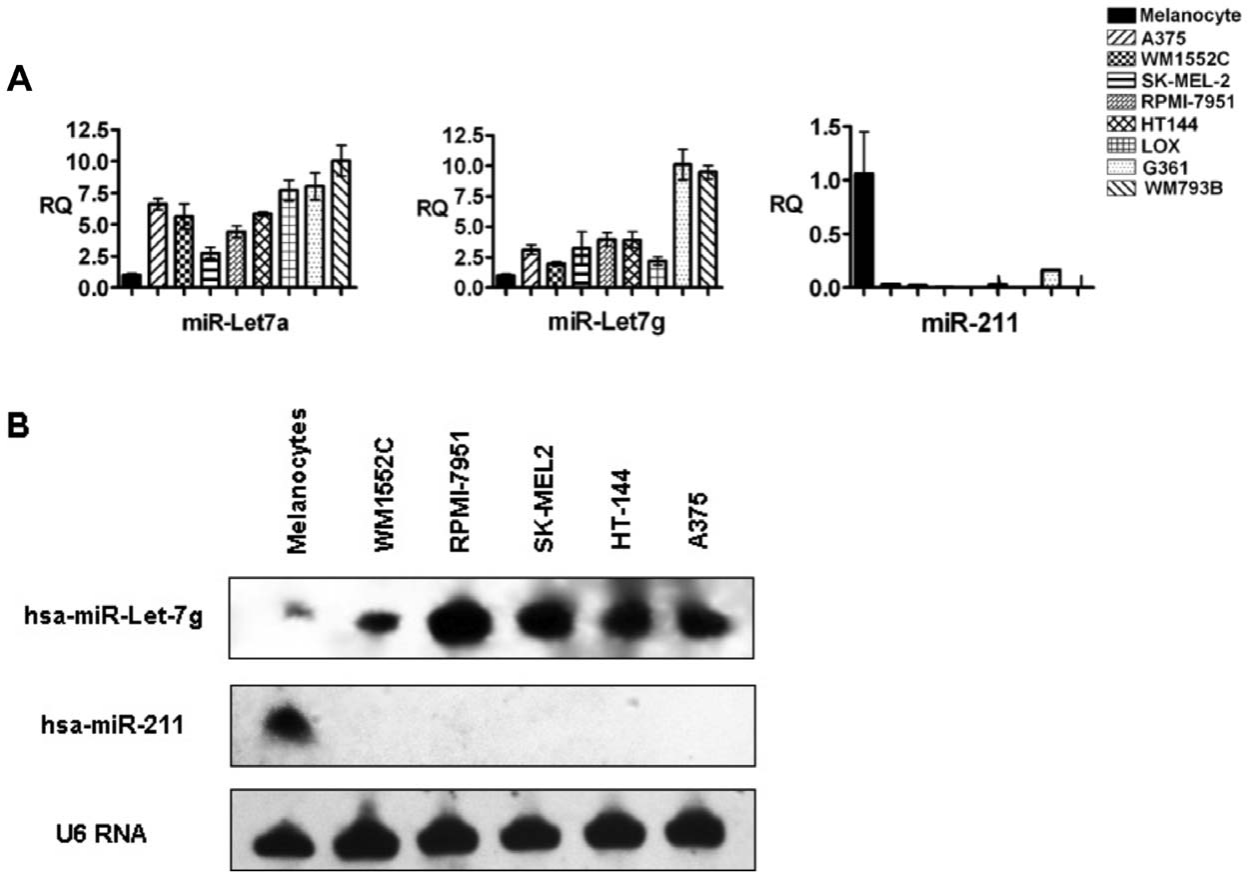
miR-211 is uniformly under-expressed in all melanoma cell lines. A) Levels of three individual miRNAs, as measured by qRT-PCR in eight different melanoma cell lines relative to their respective levels in the melanocyte cell line HEM-l, are plotted as histograms. RQ =  relative quantification index. B) Northern blot analysis of miR-211 and miR-let-7g in five melanoma cell lines and melanocytes. miR-let-7g is consistently over-expressed in melanoma cells. miR-211 is expressed at high level in melanocytes but is not detectable in any of the melanoma cell lines. Error bars are standard errors of mean of six independent measurements.

As the first step in identifying down-regulated miRNAs in human melanoma, we identified significantly differentially expressed miRNA species in the melanoma cell line WM1552C (originally established from a stage 3 skin melanoma of a 72-year-old patient) compared to those in the normal melanocyte cell line HEM-l by hybridization of total RNA samples to miRNA probe arrays (see Methods). Figure 1 lists 24 statistically significant differentially expressed miRNAs, classified into three groups according to their significance levels (P<0.01, 0.02, and 0.05, respectively). To address whether the differential miRNA expression levels observed with WM1552C varied among other established melanoma cell lines, we performed quantitative reverse transcriptase mediated polymerase chain reaction (qRT-PCR) analysis on RNA isolated from WM1552C and seven additional non-pigmented melanoma cell lines (see Methods) (Figure 2A), addressing the expression levels of three separate microRNAs: miR-let7a, miR-let7g, which were over-expressed, and miR-211 was down-regulated. Northern blot analysis further confirmed these results (Figure 2B). This consistency provided the opportunity to address the significance of the reduced level of miR-211 in melanoma. In the following sections we focus on miR-211 and its target genes as a model of the role of miRNAs that are down-regulated in melanoma, with the aim of determining the role of their target genes that are thus up-regulated in melanoma. miR-211 showed the most robust and consistent changes in expression levels between melanocytes and non-pigmented melanoma cell lines. Results reported in Figures 1 and 2 implicate several additional miRNAs in melanoma; specific studies related to these miRNAs are beyond the scope of this communication, and will be reported elsewhere.

### miR-211 levels in clinical melanoma samples

**Figure 3:**
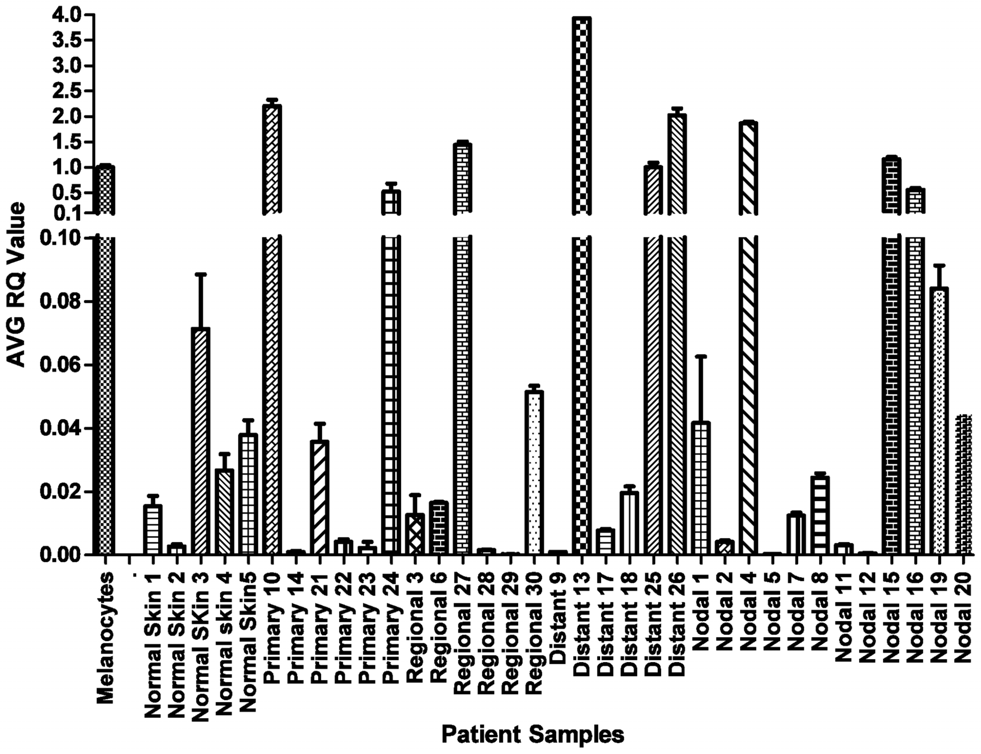
miR-211 expression in clinical melanoma samples. Histograms show normalized ratios of miR-211 levels in clinical samples relative to its level in the melanocyte cell line HEM-l (normalized to 1.0), as determined by real time quantitative RT-PCR analysis. Normal skin shows low levels of miR-211 because melanocytes constitute a small fraction of normal skin cells (see text). By two-tailed t-test, the mean RQ of miR-211 in the four groups (primary melanoma, regional, distant, and nodal metastatic melanoma) compared to the mean RQ of miR-211 in HEM-l were all statistically significant at *P*<10^−6^. MC = Melanocytes, NS = Normal Skin, PM = Primary Melanoma, RM = Regional Metastases, DM = Distant Metastases, and NM = Nodal Metastases.

We assayed miR-211 transcript levels by qRT-PCR in 30 clinical melanoma samples (six primary, six regional, 12 nodal and six distal metastatic, respectively; described in Table S1). miR-211 expression levels were reduced in 21 of these clinical samples compared to that observed in melanocytes (Figure 3, Table S2). In the remaining nine melanomas, six (one primary, one regional, two distant, and two nodal metastatic melanomas) showed statistically significant increases in miR-211 expression, whereas expression was not significantly different in the remaining samples. These samples were obtained from different patients; therefore, the observed differences may reflect different processes in melanoma development and progression, individual genetic differences, different proportions of non-melanoma (including non-pigmented) cells in the tumor samples, or a combination of these factors. Since the exact proportions of cancer cells in the frozen melanoma biopsy samples are not known, we are unable to eliminate the ratio of melanoma to non-melanoma cells as a source of the variation. Consequently, the determination of specificity and accuracy of melanoma typing by miR-211 expression were not addressed in this study. However, miR-211 levels were low in the majority (21/30) of the tested melanoma clinical samples, a statistically significant trend (*P* = 0.029, for random distribution by Fisher's exact test) that is consistent with the uniformly low expression levels in all eight melanoma-derived cell lines we studied. Note that miR-211 expression levels were also observed to be low in normal skin samples, which is expected given that melanocytes constitute a minor fraction of skin cells. Additional miRNAs that were over-expressed in melanoma cell lines relative to those in melanocytes were also over-expressed in the clinical melanoma samples but not in the normal skin samples (data not shown), confirming that normal skin samples are not the ideal background controls. Although there is no perfect “normal” counterpart tissue for melanoma in clinical skin samples, we have tested miR-211 expression levels in additional melanocyte cell lines and in five independent isolates of normal skin samples. Results show that miR-211 is elevated in both melanocyte cell lines compared to normal human skin (Figure S1). miR-211 expression levels in pooled samples of nevi also agree with previously published results, supporting the observation that miR-211 is highly expressed in nevi compared to melanoma [44]. These observations are consistent with the understanding that nevi are composed of melanocytes. Together, these results suggest that the development of most melanomas is specifically associated with the depletion of miR-211 transcript levels. An alternative formal interpretation, which is unlikely considering the absence of supporting literature, is that the low miR-211 level in melanoma reflects a cellular origin of melanoma which is distinct from that of melanocytes.

### Stable ectopic expression of miR-211 in melanoma cell lines depletes select target transcripts

**Figure 4:**
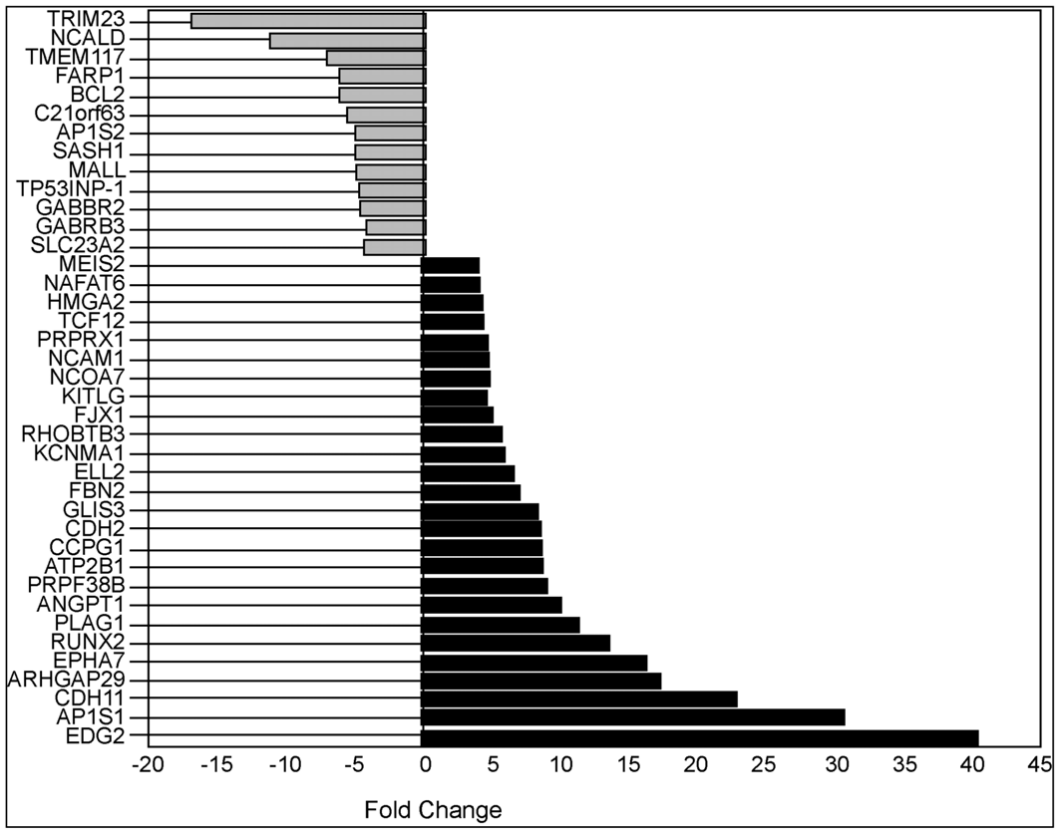
Expression of predicted target genes of miR-211 in WM1552C. Histograms of log_2_ transformed mean expression ratios (fold change) of mRNAs in WM1552C to those in the melanocyte line HEM-l are plotted. The computationally predicted target genes were selected according to criteria described in Methods. Only genes with statistically significant fold change in expression were plotted.

Differential expression of miRNAs in melanoma may be mechanistically related to melanoma development, or it may be coincidental. If indeed the depleted miRNAs are biologically relevant, melanoma cells should be enriched for their target transcripts levels relative to their corresponding levels in melanocytes. As the first step to identify such mRNA transcripts, we hybridized cDNAs made from total RNA isolated from the melanoma cell line WM1552C and the melanocyte line HEM-l to Affymetrix expression arrays. We then filtered the hybridization intensity data for differential expression of computationally predicted target transcripts of miR-211 (Figure 4) (see Methods). These experiments revealed 26 putative target transcripts whose expression levels were elevated relative to those in HEM-l.

**Figure 5:**
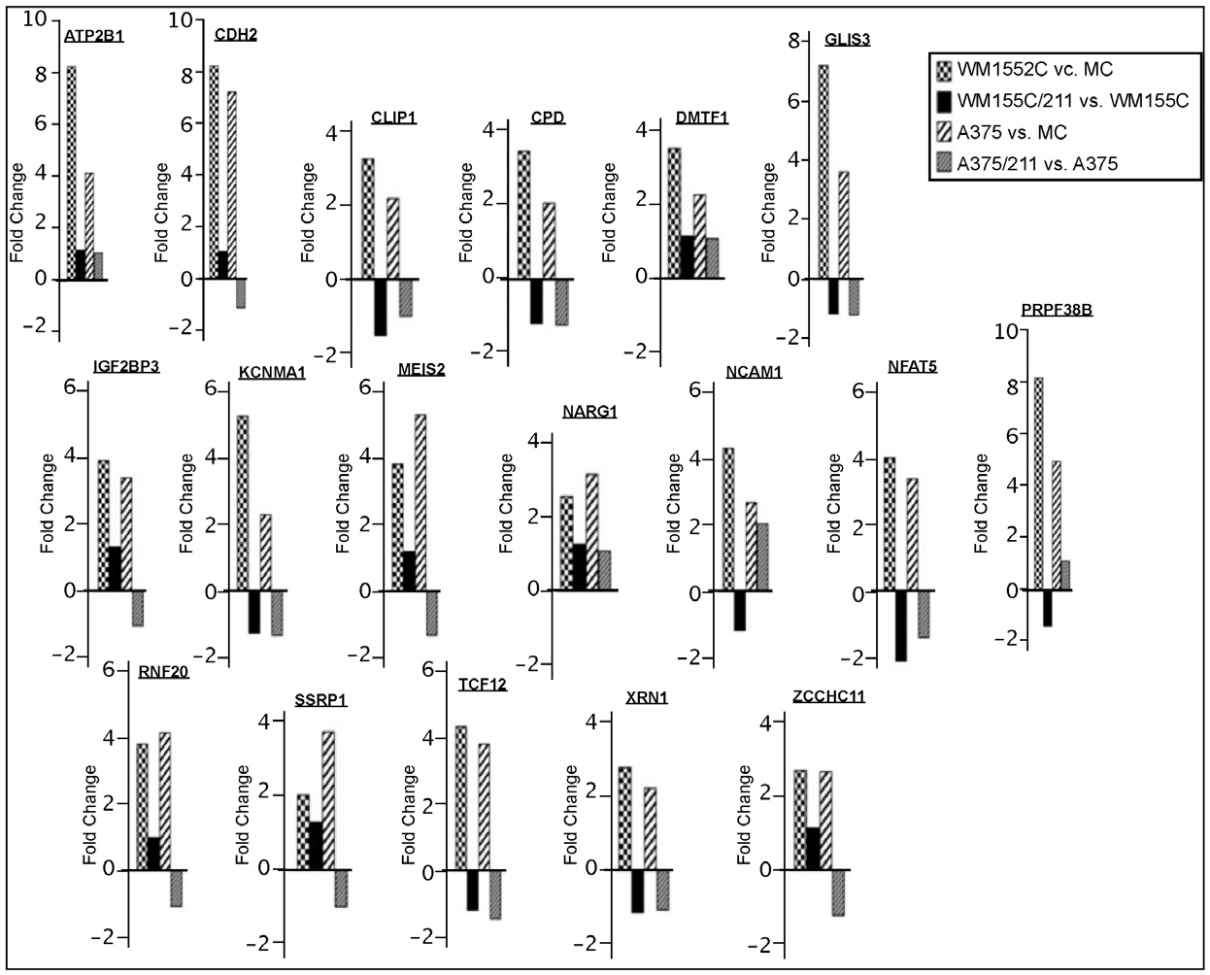
Effects of miR-211 over-expression on KCNMA1 gene expression. WM1552C and A375 melanoma cell lines were transfected with expression cassettes containing the *pre-miR-211* sequences, and stable transfectants were selected (see Methods). Expression levels of miR-211 target genes in HEM-l, A375, WM1552C, A375/211 and WM1552C/211 were measured by hybridization of cDNA (made from total RNA) to Affymetrix microarrrays. Histograms represent the log_2_ ratios of expression in different cell lines as indicated in the figure. MC = HEM-l.

If the set of 26 genes indeed contains valid targets of miR-211, their levels should be depleted if miR-211 levels were to increase in any melanoma cell line. To directly examine this possibility, we constructed three independent melanoma cell lines that stably express miR-211. For that purpose, we transfected the pre-miR-211 sequence (plasmid pcDNA4/miR-211) into WM1552C and A375 cells, followed by selection for stable expression of miR-211 and confirmation of expression by qRT-PCR analysis (see Methods). The melanoma cell line clones that ectopically expressed miR-211 were named: WM1552C/211(400), WM1552C/211(800) and A375/211 (see Methods for details). We measured global mRNA levels in WM1552C/211(400) and A375/211 cells on Affymetrix arrays and compared these levels with the corresponding levels measured in the same experiment in untransfected parental cell lines WM1552C and A375, respectively. This analysis revealed a list of 18 putative target transcripts for miR-211, which were down-regulated by the artificial expression of miR-211 in both melanoma cell lines (Figure 5). When cross-referenced with results reported in Figure 4, nine of these putative target transcripts were found to be up-regulated in both melanoma cell lines compared to those in melanocytes and down-regulated in both melanoma cell lines when miR-211 was stably expressed. These candidate targets of miR-211 are: *ATP2B1, CDH2, GLIS3, KCNMA1, MEIS2, NCAM-1, NF-AT5, PRPF38B*, and *TCF12*. Of these, the following seven genes were previously implicated in cancer progression: *ATP2B* [*45*], *CDH2* [19], [46], [47], *GLIS3* [48], *KCNMA1* [49]–[51], *MEIS2* [52], [53], *NCAM-1* [[54], and *NF-AT5* [55]. Moreover, *CDH2, KCNMA1, NCAM-1*, and *NF-AT5* were previously shown to affect metastatic migration and/or tissue invasion [19], [46], [47], [51], [54]. In particular the expression of *KCNMA1*, which encodes a component of a K^+^ exporting channel whose function is modulated by Ca^++^, has been linked to tumor cell proliferation in prostate cancer [49], cell migration in glioma [56] and antineoplastic drug resistance in melanoma cells [57]. The 3′-UTR of the KCNMA1 transcript also contains one of the strongest predicted target sites of miR-211. Therefore we focused on this transcript for investigation.

### KCNMA1 protein and transcript levels correlate inversely with that of miR-211

**Figure 6:**
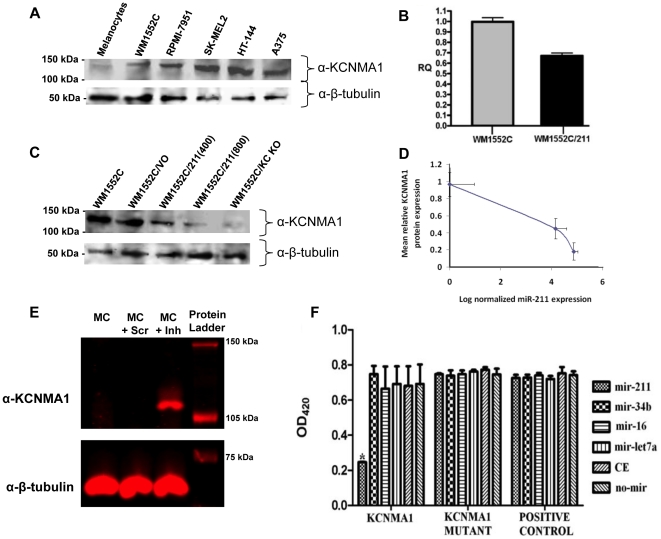
KCNMA1 mRNA is a direct target of miR-211. A) Western blot analysis of KCNMA1 protein expression in melanocytes and melanoma cell lines. Lysates were prepared from cultures of cells complementing those analyzed by northern blot in Figure 2B, including HEM-l, WM1552C, RPMI-7951, SK-MEL2, HT-144, and A375 and probed by Western blotting with antibodies against KCNMA1 or β-tubulin. B) Relative expression of *KCNMA1* mRNA in WM1552C compared to WM1552C expressing miR-211 [WM1552C/211(400)]. Histograms represent relative quantification ratio (RQ) as measured by qRT-PCR analysis. Assays were performed in triplicate. C) Western blot analysis of KCNMA1 protein expression in WM1552C stable cell lines. Lysates were prepared from cultures of untransfected WM1552C and WM1552C stably transfected with expression vectors containing: no miR (WM1552C/VO), miR-211 [both WM1552C/211(400) and WM1552C/211(800)], and an shRNA against the KCNMA1 transcript (WM1552C/KC KO), respectively, and probed by Western blotting with antibodies against KCNMA1 or β-tubulin. D) Inverse correlation between miR-211 expression and KCNMA1 protein levels. miR-211 mean RQ was measured by quantitative RT-PCR in three different strains: WM1552C/VO (normalization standard), WM1552C/211(400), and WM1552C/211(800); KCNMA1 protein levels were measured from relative fluorescence in western blots normalized against fluorescence intensity in WM1552C/VO and β-tubulin load controls. Error bars are standard errors of mean for mean RQ, and standard deviations of relative fluorescence intensity. E) Anti-miR-211 inhibitor reverses KCNMA1 protein levels in melanocytes. Melanocytes were transfected with anti-miR-211 inhibitors, and *KCNMA1* protein expression was measured in transfected cells by western blot analysis using a KCNMA1 antibody (β-tubulin was used as a load control). Derepression of KCNMA1 protein in the transfected cells is shown in the lane marked as MC+Inh. MC and MC+Scr are melanocyte controls. F) Inhibitory effect of miR-211 on mRNA containing the KCNMA1 3′-UTR sequences. An expression plasmid containing the KCNMA1 3′-UTR seed sequence for miR-211 was fused to a lacZ reporter gene (labelled, KCNMA1) such that the lacZ mRNA would contain the KCNMA1 3′-UTR sequences (harbouring the miR-211 target site) and was co-transfected into the melanoma cell line A375 with one of the following synthetic miRNAs: miR-211, miR-16-1, miR-34b, miR-let-7a, miR-CE (cel-miR-67), and no miRNA. Histograms are measurements of β-galactosidase activity at OD_420_. To directly confirm the importance of the miR-211 seed sequence, a plasmid containing the LacZ gene was fused to a mutant KCNMA1 3′UTR seed sequence (labelled, KCNMA1 Mutant), and the expression vector itself without any 3′-UTR fusion to LacZ (labelled, positive control) were also included. The assays were performed in triplicate. The only sample with statistically significant difference is indicated by an asterisk (Kruskal Wallis test, χ^2^ = 24.142, *P*<0.001).

If miR-211 targets the *KCNMA1* transcript, KCNMA1 protein expression levels should inversely correlate with that of miR-211 expression levels. A western blot analysis of KCNMA1 expression was performed, utilizing the same cell lines previously examined by northern blot (Figure 2B) for KCNMA1 transcript expression. KCNMA1 protein expression was very low in normal melanocytes, but high in all melanoma cell lines (Figure 6A), indicating an inverse correlation of expression between KCNMA1 protein and miR-211.

We next investigated whether the induced expression of miR-211 in melanoma cells can reduce KCNMA1 transcript levels. qRT-PCR analyses comparing *KCNMA1* expression in wild type WM1552C with that in WM1552C/211(400) revealed that the introduction of miR-211 down-regulates the *KCNMA1* transcript (Figure 6B). To further address whether *KCNMA1* mRNA levels reflected KCNMA1 protein expression, we performed a western blot analysis looking for KCNMA1 in cell extracts obtained from: 1) WM1552C, 2) WM1552C/VO (WM1552C cells with a stably-incorporated empty expression vector), 3) WM1552C/211(400), 4) WM1552C/211(800), and 5) WM1552C/KC KO (WM1552C cells with a stably-expressing shRNA against the *KCNMA1* mRNA) (Figure 6C). As expected, KCNMA1 protein levels were significantly reduced in both melanoma cell lines expressing miR-211 [even more so in WM1552C/211(800)] compared to those in WM1552C/VO or untransfected WM1552C cells. KCNMA1 was virtually undetectable in the WM1552C/KC KO cell line. These results are consistent with the idea that miR-211 is able to target the *KCNMA1* mRNA, thereby decreasing the amount of KCNMA1 protein in the cell. miR-211 expression was measured in engineered melanoma cell lines by qRT-PCR, and it did not exceed the levels observed in, melanocytes (Figure S2). To further confirm our observations, we measured the correlation between miR-211 expression and KCNMA1 protein levels (Figure 6D). The results revealed an inverted correlation between miR-211 expression and KCNMA1 protein levels. To confirm that this expression correlation occurred in non-transformed cells in addition to cancerous cell lines, we examined the effect of miR-211 inhibition on the expression of KCNMA1 in melanocytes. Melanocytes were transfected with anti-miR-211 inhibitors (Exiqon) and the protein expression of KCNMA1 was measured. The results indicated that derepression of KCNMA1 protein expression could be achieved by inhibition of miR-211 (Figure 6E).

### miR-211 directly targets the KCNMA1 transcript

To determine whether the computationally predicted target site of miR-211 in the 3′-UTR of the *KCNMA1* transcript confers sensitivity to miR-211, we performed a target cleavage assay with a construct containing the 3′-UTR of *KCNMA1* cDNA fused downstream of the reporter gene β-galactosidase. The construct, pcDNA6/LacZ/KCNMA1, as well as a derivative, pcDNA6/LacZ/KCNMA1-MUT (containing a mutated target cleavage site at the seed sequence; see Figure S3), and the control vector pcDNA6/LacZ, were separately transfected into A375 cells along with one of the following miRNA mimics: miR-211, miR-16-1, miR-34b, miR-let-7a-1, cel-miR-67, or no mimic (Figure 6E). The results revealed a statistically significant drop of nearly 60% in β-galactosidase activity when the cells were transfected with pcDNA6/LacZ/KCNMA1 together with miR-211 mimics, but not with any other combination. Importantly, this drop was not detectable in cells co-transfected with pcDNA6/LacZ/KCNMA1-MUT and the miR-211 mimic, demonstrating that miR-211 was capable of specifically targeting the wild type seed sequence in the 3′-UTR of the *KCNMA1* transcript.

### MITF co-ordinately regulates *miR-211* and *TRPM1*

**Figure 7:**
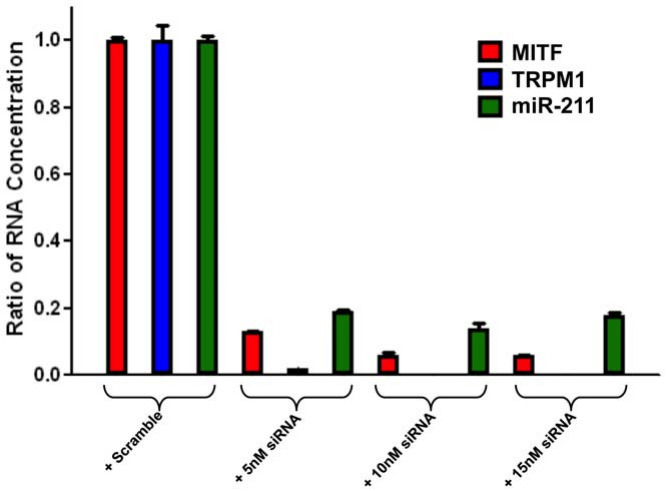
The effect of MITF knock-down on TRPM1 and miR-211 expression in pigmented melanoma cells; SKMEL-28. Relative gene expression levels of MITF, TRPM1, and miR-211 in MITF knock-down cells. Three different doses of MITF siRNA (5 nM, 10 nM and 15 nM) was used to knock-down MITF gene and expression values are normalized to scramble siRNA control. Histograms represent the Ratio of RNA Concentration, as measured by qRT-PCR analysis.

The gene encoding miR-211 is located within the sixth intron of the *TRPM1* gene, which encodes multiple polypeptide isoforms including melastatin-1, a transient receptor potential (TRP) protein family member thought to be a potential suppressor of melanoma metastasis [58]. However, the molecular basis of the tumor suppressor activity of *TRPM1* gene is not understood. The transcription factor MITF regulates the expression of *TRPM1* gene, where the MITF-binding motif (GCTCACATGT) is located in the *TRPM1* promoter [58]. This prompted us to examine whether MITF also might transcriptionally regulate miR-211 expression via the *TRPM1* promoter. We found that both TRPM1 and miR-211 transcripts are expressed in pigmented but not in the non-pigmented melanoma cells. To determine whether MITF expression modulates miR-211 expression, we knocked down MITF expression by siRNA in the pigmented melanoma cell line SK-MEL28. Three different doses of siRNA (5 nM, 10 nM and 15 nM) were used, and the knock-down efficiency was measured by qRT-PCR. As expected, the extent of reduction in MITF transcript levels directly correlated with the reduction in TRPM1 and miR-211 transcript levels (Figure 7). In conclusion, the results are consistent with the hypothesis that MITF co-ordinately regulates TRPM1 and miR-211 expression. If true, it raises the possibility that one of the ways MITF might also suppress melanoma metastasis is through its transcriptional activation of miR-211 via the *TRPM1* promoter, and the consequent negative post-transcriptional effects of miR-211 on KCNMA1 mRNA.

### The effect of miR-211 on cell proliferation and invasion

**Figure 8:**
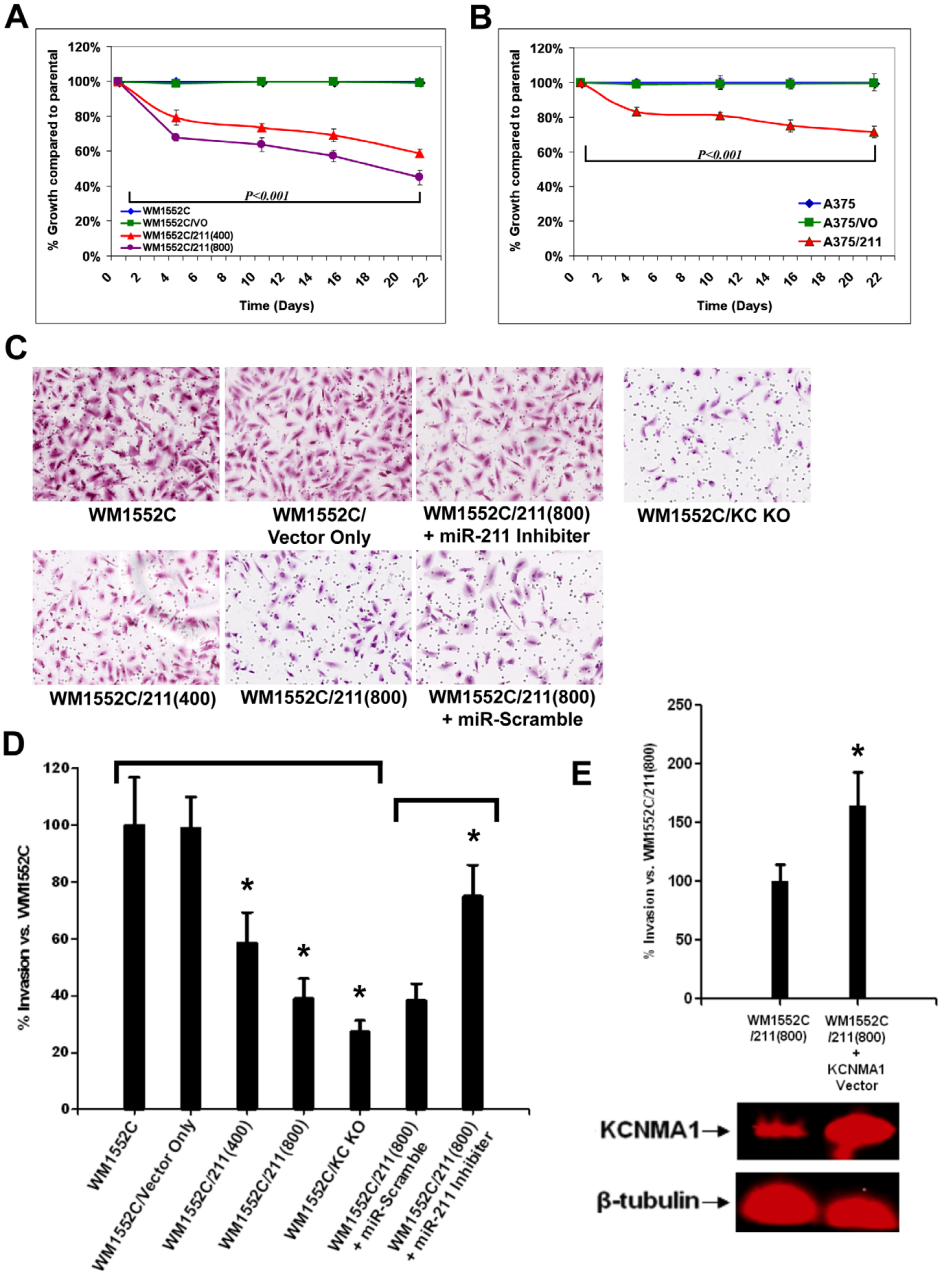
Effects of miR-211 over-expression on melanoma cells. A and B: Relative mean cell titers of (A) WM1552C/211(400), WM1552C/211(800), and WM1552C/VO (vector only) cells to that of WM1552C cells and of (B) A375/211(400) and A375/VO (vector only) cells to that of A375 cells. (C) and (D): Cell invasion assays comparing WM1552C to WM1552C/VO, WM1552C/211(400), WM1552C/211(800), and WM1552C/KC KO stable derivatives, as well as to WM1552C/211(800) transfected with the Anti-miR miRNA Inhibiter for hsa-miR-211 [labelled “WM1552C/211(800) + miR-211”] or Negative Control #1 (labelled “WM1552C/211(800) + miR-Scramble”) over 48 hours. Each assay was performed in triplicate. Statistical significance is indicated by an asterisk in the figure pertaining to the experimental group delimited by a bar over the histograms (*P-value*<0.001). E) The artificial expression of KCNMA1 protein in WM1552C/211(800) cells increases melanoma cell invasiveness. Western blot results show that KCNMA1 protein levels are elevated in transfected cells [“WM1552C/211(800) + KCNMA1 Vector” relative to control cells without KCNMA1 expression vector] (bottom). β-tubulin was used as a load control. Results from the invasion assay illustrate that the KCNMA1 protein expression increased melanoma cell invasiveness (top).

Since the over-expression of *KCNMA1* is often associated with both cell proliferation and cell migration/invasion in various cancers [49]–[51], we decided to determine whether the depletion of miR-211 and associated over-expression of *KCNMA1* were important for these processes in melanoma cells. We began by comparing the proliferation rates of melanoma cell lines stably transfected with the miR-211 expression cassette with those of untransfected melanoma cells and cell lines transfected with the empty expression vector (Figure 8A) (see Methods), respectively. All miR-211-expressing cultures of WM1552C/211 showed reduced cell counts compared to those of WM1552C beginning at even the first time point (day 4), and the titre continued to fall behind as time progressed. After a 21-day period, WM1552C/211(400) had greater than 30% decrease in cell counts compared to those of WM1552C, while WM1552C/211(800) cultures showed an even greater decrease in cell proliferation. WM1552C/VO cells showed no significant difference in cell proliferation compared to WM1552C. Comparable results were obtained for cell proliferation of A375/211 cell lines, which grew more slowly than untransfected A375 or A375/VO (Figure 8B). These results are consistent with the hypothesis that an important growth stimulatory event in the melanoma cell lines WM1552C and A375 involves the depletion of miR-211 levels—the latter possibly leading to the targeted up-regulation of at least *KCNMA1* expression among its target genes.

Next, we examined the impact of miR-211 expression on the invasive properties of WM1552C. WM1552C/211(400) and WM1552C/211(800) cells, along with WM1552C/VO, WM1552C/KC KO, and untransfected WM1552C cells were seeded separately into invasion chambers, and the cells were allowed to migrate (see Methods). Results indicated that WM1552C/211(400) and WM1552C/211(800) cells migrated significantly less (∼40% and 60% less, respectively) than WM1552C (Figures 8C and 8D), whereas WM1552C/VO cells showed almost no variation compared to parental cells. The frequency of cells with invasion defects significantly exceeded the decrease in the proliferation rates of these cells (an ∼8–10% decrease in growth over the 48 hours of the invasion assay period), suggesting that the two effects on miR-211 expression are independent of each other. The most significant effect on invasion was observed in the WM1552C/KC KO cells. While a sequence-scrambled oligonucleotide (miR-Scramble) did not show an effect on cell invasion, cells treated with a miR-211 inhibitor restored the invasion phenotype by as much as 40% (Figure 8D). Given that previously published evidence directly links *KCNMA1* gene dosage and/or expression with increased motility/invasion in several cancers [49]–[51], these results suggest that at least part of the invasion defect caused by miR-211 in melanoma cell lines is due to targeted down-regulation of the KCNMA1 transcript. To fully demonstrate that KCNMA1 is a key contributor to miR-211 effects, we examined whether concomitant over-expression of KCNMA1 might also rescue the miR-211 anti-invasive effects. A KCNMA1 constitutively-expressing plasmid was transiently transfected into WM1552C/211(800) cells. This plasmid (Origene clone NM_002247.2) contains a KCNMA1 ORF without its native 3′UTR (making it resistant to regulation by miR-211). KCNMA1 protein expression levels were then detected by KCNMA1 antibody. Western blot results revealed that KCNMA1 protein levels were elevated in transfected cells [“WM1552C/211(800) + KCNMA1 vector” relative to control cells] (Figure 8E, bottom). Results from an invasion assay (Figure 8E, top) illustrate that the same batch of melanoma cells that exhibit high KCNMA1 protein expression [WM1552C/211(800) + KCNMA1 vector” cells] also show high cell invasiveness, higher by at least 60% compared to the control cell cultures.

## Discussion

Current understanding of the molecular mechanisms of carcinogenesis is beginning to include not only the role of protein coding genes but also that of non-coding regulatory RNA, especially miRNAs. In the case of melanoma, our discovery of miRNAs whose expression levels are reduced in melanoma cells can potentially lead to the identification of genes that are responsible for oncogenesis and invasiveness. Along that line, we report here that miR-211 levels are consistently reduced in melanoma cells compared to its levels in melanocytes, and that the expression levels of several potential miR-211 target mRNAs are elevated in melanoma cells. We demonstrate that the increased expression of one particular confirmed target transcript, KCNMA1, is associated with high invasiveness and proliferation in melanoma cells *in vitro*.

The simplest model we offer is that the down-regulation of miR-211 causes elevated levels of KCNMA1 protein in melanoma cells, which at least in part explains the invasiveness of malignant melanoma. More complex models are possible, such as yet unidentified targets of miR-211 (besides KCNMA1) that may have a positive feedback effect on KCNMA1 levels and are responsible for invasiveness. Another alternative possibility is that miR-211 down-regulation in melanoma causes other transformational events unrelated to *KCNMA1*, leading to higher oncogenesis and invasiveness. Both of these more complex possibilities are consistent with some of our results, but not with the full set of results presented here. While the assays of cell invasion reported here are widely used for demonstrating metastatic potential [59], [60], and the results appear convincing, further *in situ* studies with immunodeficient mice are needed to confirm the role of *KCNMA1* in melanoma invasiveness *in vivo*. We observed that melanoma cell lines engineered to express high levels of miR-211 begin to lose expression shortly after removal from selection, indicating a strong bias against miR-211 expression during the growth of melanoma cell lines and suggests that the rapid proliferation of melanoma cells in culture is directly related to low miR-211 activity in these cells. Future experiments will explore whether the progressive reduction in miR-211 levels observed in these engineered cells is due to genetic or epigenetic changes.

**Figure 9:**
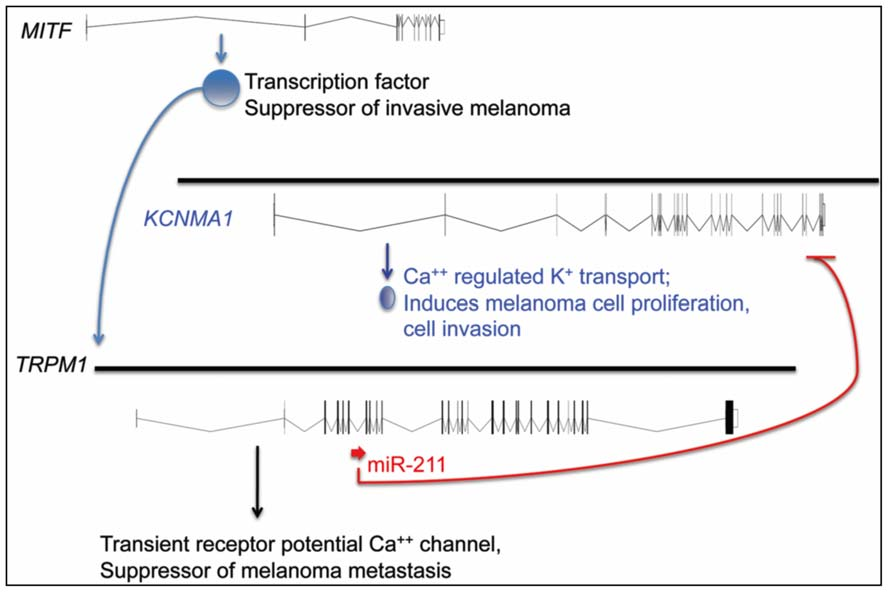
A model summarizing the regulation and role of miR-211 in melanoma. MITF, a transcription factor with tumor-suppressor activity, is active in melanocytes, where it is required for the expression of *TRPM1*, the structural gene for melatonin-1. *miR-211* gene is located within the sixth intron of *TRPM1*, which is co-transcribed with the *TRPM1* transcript, is processed to active miR-211 and subsequently the latter acts on the 3′-UTR of KCNMA1 transcript. We propose that inhibition of KCNMA1 translation is needed for preventing the development of invasive melanoma. MITF activity is low in melanoma cells, which is expected to reduce *TRPM1* as well as *miR-211* transcription, therefore would induce the expression of *KCNMA1*. While it is widely held on the basis of expression pattern alone that *TRPM1* is a putative tumor suppressor, we show that miR-211, contained within the pre-mRNA of TRPM1 transcript, also has a tumor suppressor activity through its negative regulation on KCNMA1.

The *TRPM1* gene, which contains *miR-211* sequences in the sixth intron, was previously suggested to be a suppressor of melanoma aggressiveness [61], [62]. We showed here that the transcription factor MITF, which regulates the expression of *TRPM1*, is also needed for high-level expression of *miR-211*. Thus, the regulation by MITF of both *TRPM1* and *miR-211* genes can be speculated to have similar effects on melanoma invasiveness separately through their respective gene products: the former a Ca^++^ channel protein (TRPM1), and the latter a miRNA targeted against the Ca^++^ regulated K^+^ channel protein KCNMA1. If true, the invasiveness of melanoma cells could partly be the result of the breakdown of processes related to calcium-regulated ion homeostasis. The recent finding that salinomycin, an inhibitor of K^+^ transport, is a selective inhibitor of cancer stem cell proliferation is consistent with our findings on the role of KCNMA1 in melanoma cells [63]. We cannot eliminate the formal possibility that the potential tumor suppressor activity of *TRPM1* gene is, at least in part, due to the co-expression of miR-211 encoded from within its sixth intron. In Figure 9 we summarize our results, in light of previous studies, as a simple model of the mechanism of development of invasive melanoma, which highlights the role of miR-211.

In contrast to our finding that miR-211 levels in most melanoma cells and clinical samples were down-regulated, Gaur *et al.* [64] previously reported that miR-211 was over-expressed in 6 of 8 tested melanoma lines from the NCI-60 panel of cancer cells. However, a leave-one-out sensitivity analysis conducted by Gaur *et al*. [64] failed to show a significant effect on the confidence interval when miR-211 expression level was omitted, suggesting low specificity or sensitivity with respect to miR-211 in those experiments. Muller *et al*. [41] compared miRNA expression in melanoma cell lines with pooled normal human epidermal melanocytes; miR-211 was not down-regulated in their study. It is likely that the melanocyte cells (pooled epidermal melanocytes) these authors used were physiologically and genetically different from the melanocyte lines we used. Jukic *et al.*, [44] reported that miR-211 was up-regulated in nevi and dramatically down-regulated in metastatic melanoma compared to nevi controls. These results are in agreement with our results and contradict the results published by Schultz, et al.,[31].

In conclusion, we have demonstrated that miR-211 is down-regulated in non-pigmented melanoma and its expression is regulated by the MITF gene. The down-regulation of miR-211 and the corresponding up-regulation of its target transcript KCNMA1 are therefore important molecular events for melanoma development and/or progression.

## Methods

### Cell lines and clinical samples

The human epidermal melanocyte cell line HEM-l (ScienCell™, Catalog # 2200) and primary epidermal melanoyctes –neonatal (ATCC - PCS-200-012) were grown in MelM media containing MelGS growth supplements, 0.5% FBS, and pen/strep solution. The melanoma cell lines examined included: A375 (stage 4, ATCC® Number: CRL-1619), G361 (stage 4, ATCC), LOX-IMV1 (stage 4, ATCC), HT-144 (stage 4, ATCC® Number: HTB-63), RPMI-7951 (stage 4, ATCC® Number: HTB-66), SK-MEL2 (stage 4, ATCC), SK-MEL28 (stage 3, ATCC), WM793B (stage 1, ATCC® Number: CRL-2806), and WM1552C (stage 3, ATCC® Number: CRL-2808). All melanoma cell lines were grown in Complete Tu Media containing a 4∶1 mixture of MCDB-153 medium with 1.5 g/L sodium bicarbonate and Leibovitz's L-15 medium with 2 mM L-glutamine, 2% FBS, and 1.68 mM CaCl_2_.

Information regarding all clinical samples, derived from frozen samples, is described in Table S1.

### miRNA arrays

miRNA NCode™ version 2 array (Invitrogen) containing 553 human and 427 mouse miRNAs, and the TILDA array (ABI) were used for miRNA expression profiling. The miRNA samples were labelled with AlexaFluor® conjugated dendrimers using the direct labelling kit (Genisphere). We routinely evaluated hybridization conditions by discriminating between 2 nt variants at internal sites, and most probes can distinguish between 1 nt variants. The arrays were scanned with Axon B-4000 (Agilent).

### Validation of miRNA array results

Expression levels of all statistically significant and differentially expressed mRNAs and miRNAs were confirmed by qRT-PCR using TaqMan® expression kits (Applied Biosystems) [65] using multiple technical and biological replicates. *GAPDH* was used as the internal reference probe for normalization of expression values of mRNA, and *RNU48* was used for normalization of miRNA. RNA analysis by Northern blots used 20 µg of total RNA concentrated from each sample (melanoma cell lines and melanocytes), separated on 15% urea denaturing polyacrylamide gels by electrophoresis. Gels were electroblotted to nylon membranes, cross-linked by UV, prehybridized in ULTRAhyb®-Oligo (Ambion) for 30 minutes at 42°C, and hybridized with 5′-biotinylated anti-miRNA DNA oligonucleotides (100 nM each) at 42°C overnight, washed, and detected by chemiluminescence (Brightstar® detection kit, Ambion). Anti-U6 probes were used as a reference control (at 10 pM).

### Microarray data analyses and miRNA target prediction

For the initial transformation of miRNA array data, the GenePixPro 6.0 global normalization method was employed in which images and results are normalized together. Statistical significance tests were Welsh t-test, nonparametric ANOVA, (*e.g.*, to select genes that have significantly less within sample variance compared to between sample variance), and correlation analysis with Pearson's product moment **r** and Spearman's ***r***. Analysis was controlled for false discovery rate using q-values, with *a priori* cut off point of 10 percent [66], [67]. For mRNA expression array data, commencing with GeneChip® Human Exon 1.0 ST Array (Affymetrix, Inc.) four probes per exon and roughly 40 probes per gene, 7 total arrays were analysed (three arrays for melanocyte RNA, and four arrays for melanoma RNA). Cell files were loaded into Partek® Genomics Suite™ (Partek, Inc. St. Louis, Missouri, USA) under the following algorithm constraints: interrogating probes selection, RMA background correction, adjusted for GC content, quintile normalization, log probes using base 2, with probe set summarization of median polish. Quality control assessment indicated clear separation based on the cell type. Gene level analysis use an ANOVA model; y_j_ = µ+T_j_+€, where µ is the mean expression of the gene, T_j_ is the tissue type, and € is the error term. The ANOVA model generated a significance level for each probe set, along with the fold change, and imputed gene annotations. miR-211 target set of genes were obtained from public databases [miRanda, miRbase, miRNAmap, Tarbase, PicTar, Target ScanS, and DIANA MicroTest (http://www.ncrna.org)], and the results from ANOVA were matched to obtain the final target gene list of genes. This target list was imported into Ingenuity Pathway Analysis Version 6.0-1202 (Ingenuity Systems®). A core analysis was run employing direct relationships only, the Ingenuity knowledge base genes as the reference set, and with down-regulators as the defined expression value parameter. All microarray data have been deposited into GEO, and accession number is pending.

### Construction of miR-211 expression plasmids

Oligonucleotides complementary to the *miR-211* genomic sequences (miR-211 pre For – ttccctttgtcatccttcgcct and miR-211 pre Rev – aggcgaaggatgacaaagggaa, containing *Hin*dIII and *Bam*HI sites on their respective 5′ and 3′ ends) were used to amplify the 110 bp *pre-miR-211* sequence from human melanocyte genomic DNA (Amplitaq Gold®, Applied Biosystems) and TOPO®-cloned into the pCR®4-TOPO® vector (Invitrogen). The construct was sequenced, and the pre-hsa-miR-211 fragment was sub-cloned into pcDNA4/myc-HisA (Invitrogen) to create pcDNA4/miR-211. The *KCNMA1* siRNA sequence was derived from Silencer® siRNA (Ambion, siRNA ID: 112882) and constructed as long complementary oligos (KCNMA1si For – cgtacttcaatgacaatatttcaagagaatattgtcattgaagtacgtctttttt and KCNMA1si Rev – aaaaaagacgtacttcaatgacaatattctcttgaaatattgtcattgaagtacg, containing *Hin*dIII and *Bam*HI sites on their respective 5′ and 3′ ends). The oligos were mixed at 100 µM, heated, and amplified through one round of PCR (Amplitaq Gold®, Applied Biosystems) and then TOPO®-cloned into the pCR®4-TOPO® vector (Invitrogen). Inserts were sequenced and then sub-cloned into pcDNA4/myc-HisA (Invitrogen) to create pcDNA4/shKCNMA1.

### Construction of stable melanoma cell lines

2.5×10^5^ WM1552C or A375 melanoma cells were seeded into a single well of a 6-well plate and transfected overnight with 5 µg pcDNA4/miR-211, pcDNA4/shKCNMA1, or pcDNA4/myc-HisA (“vector only” negative control) using Fugene® 6 (Roche). The transfected cells were selected at 400 or 800 µg/mL Zeocin™ for 15 days, and the presence of the transgene copy in stable Zeocin™-resistant foci was confirmed by PCR (Amplitaq® Gold, Applied Biosystems). Cell lines were named WM1552C/211(400) or A375/211(400) when selection was at 400 µg/ml Zeocin™, and WM1552C/211(800) when selection was at 800 µg/ml Zeocin™, respectively. The “vector only” control cells were selected at 800 µg/ml Zeocin™. WM1552C/KC KO were selected at 400 µg/ml Zeocin™.

### Target cleavage assays

The 3′ UTR seed sequences of putative target genes were amplified by PCR (Phusion™ PCR kit, Finnzymes) from human melanocyte genomic DNA (Primers: KCNMA1 For – tgcggccgccttccctatatctaaacaatgcaaaatc, KCNMA1 Rev – aaccggtcacccatccaggcgaggagc, the primer set contained 5′ *Not*I or 3′ *Age*I sites). The PCR product was cloned into pCR®4-TOPO® (Invitrogen), confirmed by sequencing, then sub-cloned into the 3′ UTR of the LacZ gene in pcDNA6/V5-His/LacZ (Invitrogen) using the 5′ *Not*I and 3′ *Age*I restriction sites and reconfirmed by sequencing (pcDNA6/LacZ/KCNMA1). The cloned 3′UTR of KCNMA1 was mutated using the primers: KC Mut For- TACGCATATGAATTATTAAAACAATTTT and KC Mut Rev - TATGCGTAAATTACAATTAATTGTGCT, and used to PCR amplify pcDNA6/LacZ/KCNMA1 using Quickchange (Stratagene). The plasmid product was then recovered and confirmed by sequencing (pcDNA6/LacZ/KCNMA1-MUT, see Figure S4 for mutagenesis). A375 melanoma cell lines were then transfected in triplicate (Fugene® 6, Roche) with 5 µg plasmid DNA of: A) pcDNA6/LacZ/KCNMA1, B) pcDNA6/V5-His/KCNMA1-MUT or C) pcDNA6/V5-His/LacZ (positive control), and co-transfected (siPORT™, Ambion) at 100 nM with miRIDIAN microRNA Mimics (Dharmacon) for A) miR-16-1, B) miR-211, C) miR-34b, D) miR-let-7a-1, E) miRIDIAN cel-miR-67 (negative control; cel-miR-67 has been confirmed to have minimal sequence identity with miRNAs in human, mouse, and rat), or F) no mimic miRNA. After overnight incubation, cells were washed in PBS and reincubated in fresh media. After 48 hours, cells were harvested by trypsinization, examined for viability, and samples were prepared for the β-galactosidase assay using the β-Gal Assay kit (Invitrogen). Samples were incubated overnight at 37°C, then assayed for β -galactosidase activity in a 96-well plate format using a FlexStation3 (Molecular Devices).

### Western blot analysis of KCNMA1

Total lysates of 5×10^5^ cells of each cell line were boiled under denaturing conditions and proteins separated on 6% Tris-Glycine denaturing polyacrylamide gels by electrophoresis. Proteins transferred to nitrocellulose membranes were probed with the following primary antibodies: anti-Slo1 (NeuroMab, UC Davis) at 1/500 and anti-β-tubulin (BD Pharmingen) at 1/2000 according to standard methods. Blots were probed with horseradish peroxidase-conjugated secondary antibodies and visualized with ECL chemiluminescence (Pierce) or Alexa 680-conjugated secondary antibodies (Molecular Probes) and visualized on the Licor Odyssesy (Licor).

### Growth rate assays for miR-211 stable melanoma cell lines

Assays were performed using WM1552C, WM1552C/VO, WM1552C/211(400), WM1552C/211(800), A375, A375/VO, and A375/211 cell lines. Cells were grown in log phase, trypsinized, counted using an automated cell counter (Cellometer®, Nexcelom Bioscience), and then seeded into 75 cm^2^ flasks at 5×10^5^ cells per flask (in triplicate). Media was changed after 6 hours, and cells were further fed every 48 hours (Complete Tu Media). At days 4, 10, 15, and 21, cells were trypsinized, counted (Cellometer®, Nexcelom Bioscience), and then reseeded. Each assay was performed in duplicate for all cell lines.

### Invasion assays

BD BioCoat™ growth factor reduced insert plates (Matrigel™ Invasion Chamber 12 well plates) were prepared by rehydrating the BD Matrigel™ matrix coating in the inserts with 0.5 mls of serum-free Complete Tu media for two hours at 37°C. The rehydration solution was carefully removed from the inserts, 0.5 ml Complete Tu (2% FBS) was added to the lower wells of the plate, and 2.5×10^4^ cells suspended in 0.5 ml of serum-free Complete Tu media were added to each insert well. WM1552C/211(800) cells were additionally transfected with the Anti-miR miRNA Inhibiter for hsa-miR-211 as well as Negative Control#1 (Ambion) (miR-Scramble) at a concentration of 100 nM using siPORT NeoFX (Ambion). Invasion assay plates were incubated for 48 hours at 37°C. Following incubation, the non-invading cells were removed by scrubbing the upper surface of the insert. The cells on the lower surface of the insert were stained with crystal violet, and each trans-well membrane was mounted on a microscope slide for visualization and analysis. The slides were scanned using the Aperio Scanscope XT and visualized using the Aperio Imagescope v10 software. The number of migrating tumor cells was counted from each of five images per cell line (including miR Inhibiter transfected cells) in the central area of the filter. Cell lines were tested in triplicate, and the assays were performed twice. Data are expressed as the percent invasion through the membrane relative to the migration of WM1552C (Wild Type) through the membrane.

### Transfection of Human Melanocytes using Anti-miR-211 Inhibiter molecules

5×10^5^ HEM-l cells were seeded into wells of a 6-well plate. The cells were then transfected with Fugene® 6 (Roche) and either 100 nM of anti-miR-211 Inhibitors (Exiqon), 100 nM of anti-miR Inhibiter Negative Control #1 (“miR-Scramble”), or transfection agent only. After 48 hours, the cells were harvested by trypsinization and counted using an automated cell counter (Cellometer®, Nexcelom Bioscience). 2.5×10^5^ cells were then prepared for western blotting (as above).

### Transient Expression of KCNMA1 in a stable miR-211-expressing melanoma cell line

2.5×10^5^ cells WM1552C/211(800) cells were seeded into wells of a 6-well plate. 1 well was transfected with 5 µg of KCNMA1-expressing plasmid (Origene catalog # SC122078) using Fugene® 6 (Roche) and a second well was treated with transfection reagent only. After 48 hours, the cells were harvested by trypsinization and counted using an automated cell counter (Cellometer®, Nexcelom Bioscience). 2.5×10^4^ cells were then utilized for invasion assays (in triplicate) and 2.5×10^5^ cells were prepared for western blotting (as above).

## Supporting Information

Figure S1 miR-211 expression in melanocytes, normal skin and nevus. Both melanocytes and nevus sample indicate a higher expression of miR-211. Melanocyte A - HEM-l, Melanocyte B - HEM (neonatal cell line).
(0.26 MB TIF)

Figure S2 miR-211 expression in stable melanoma cell lines compared to melanocytes. Two stable miR-211-expressing WM1552C cell lines, as well as a “Vector Only” (VO) control cell line, as measured by qRT-PCR relative to levels in the melanocyte cell line HEM-l, are plotted as histograms. Error bars are standard errors of mean of three independent measurements.
(0.24 MB TIF)

Figure S3 Mutagenesis of miR-211 target seed sequence in the 3′UTR of KCNMA1. Diagram indicates the four nucleotides altered in the target seed sequence within the 3′UTR of KCNMA1 relative to the wild type.
(0.25 MB TIF)

Table S1 Description of human clinical samples.
(0.72 MB TIF)

Table S2 miR-211 expression levels in clinical samples. Note: Two-tailed t-test comparisons for miR-211 by mean relative quantification levels of melanocyte and primary melanoma, as well as regional, distant, and nodal metastatic melanoma were all statistically significant at P<0.000001.
(0.84 MB TIF)
