# Supplementary figures and images for: The Regulation of miRNA-211 Expression and Its Role in Melanoma Cell Invasiveness
Source: PLoS One. 2010 Nov 1;5(11):e13779. doi: 10.1371/journal.pone.0013779 (PMC2967468; doi:10.1371/journal.pone.0013779)

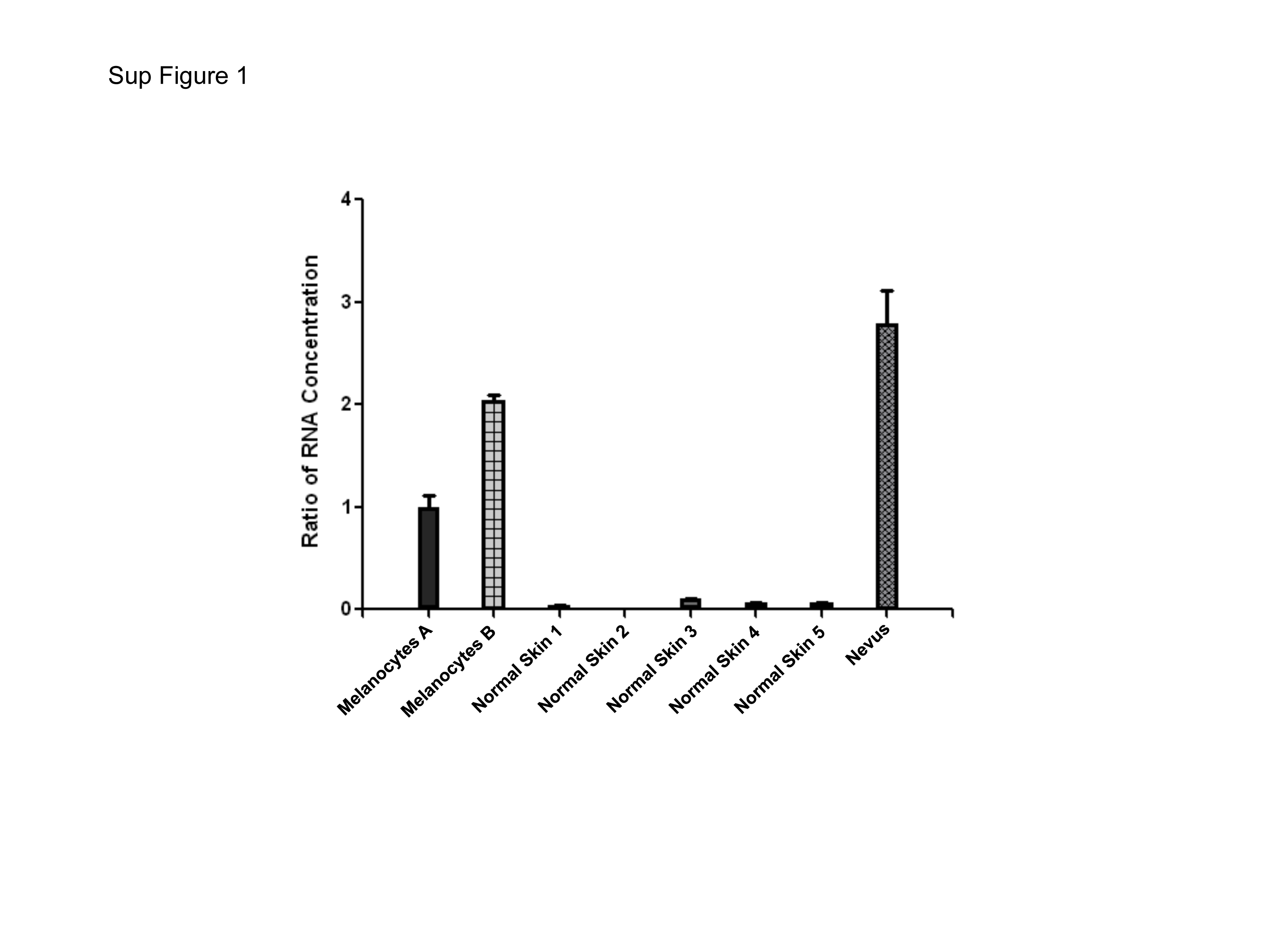

Supplement: Figure S1 — miR-211 expression in melanocytes, normal skin and nevus. Both melanocytes and nevus sample indicate a higher expression of miR-211. Melanocyte A - HEM-l, Melanocyte B - HEM (neonatal cell line). (0.26 MB TIF) [file pone.0013779.s001.tif]

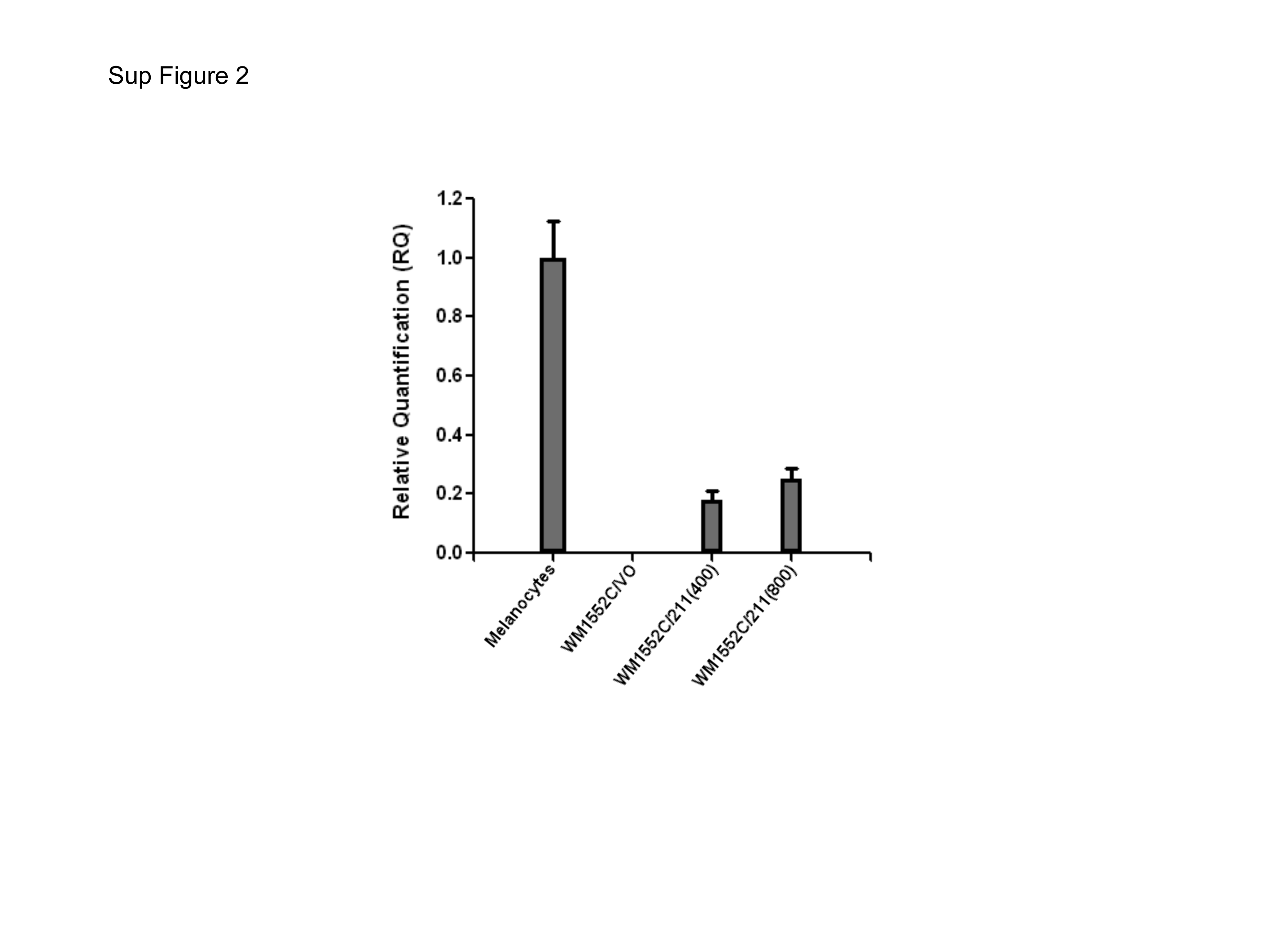

Supplement: Figure S2 — miR-211 expression in stable melanoma cell lines compared to melanocytes. Two stable miR-211-expressing WM1552C cell lines, as well as a “Vector Only” (VO) control cell line, as measured by qRT-PCR relative to levels in the melanocyte cell line HEM-l, are plotted as histograms. Error bars are standard errors of mean of three independent measurements. (0.24 MB TIF) [file pone.0013779.s002.tif]

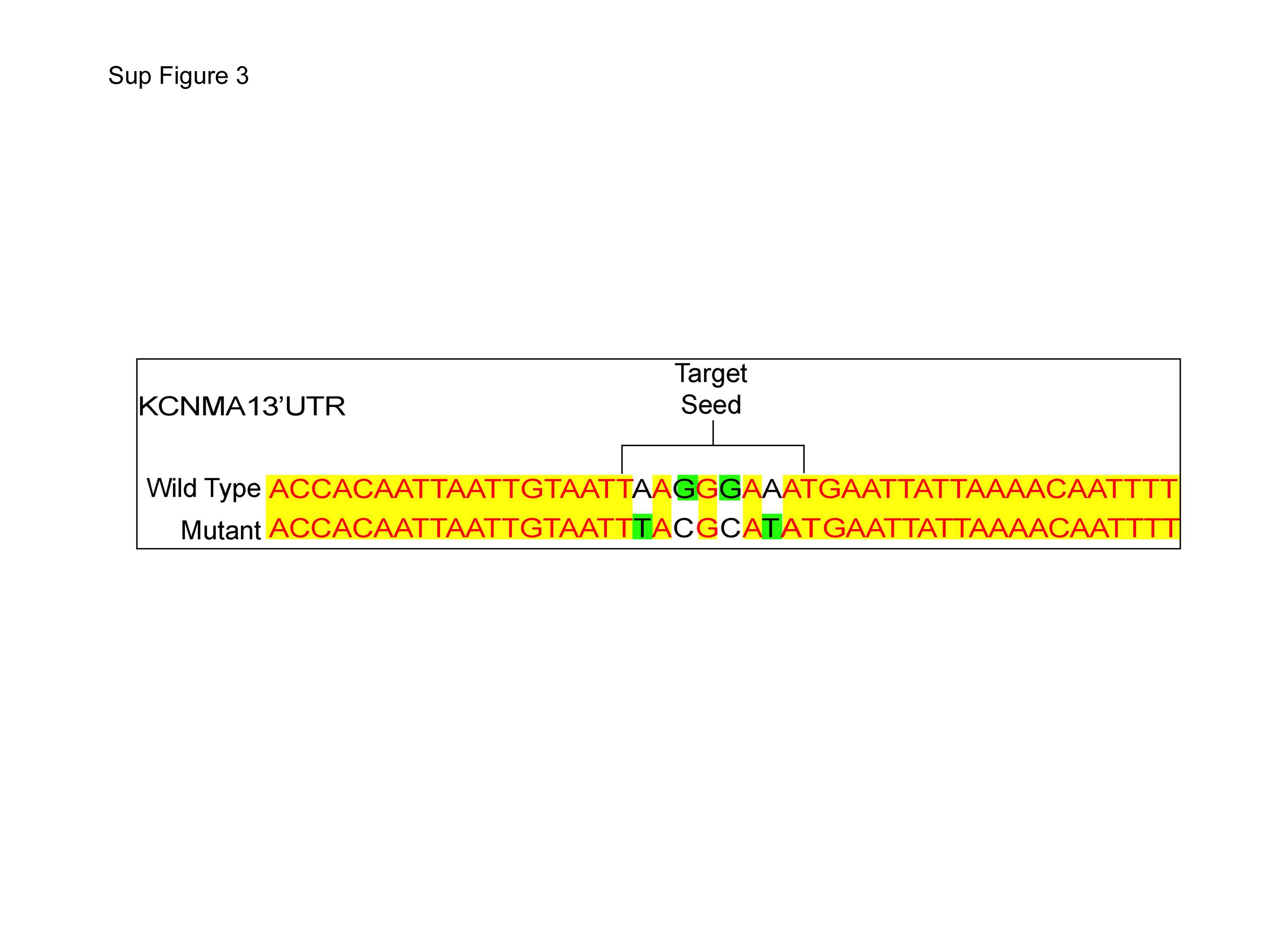

Supplement: Figure S3 — Mutagenesis of miR-211 target seed sequence in the 3′UTR of KCNMA1. Diagram indicates the four nucleotides altered in the target seed sequence within the 3′UTR of KCNMA1 relative to the wild type. (0.25 MB TIF) [file pone.0013779.s003.tif]

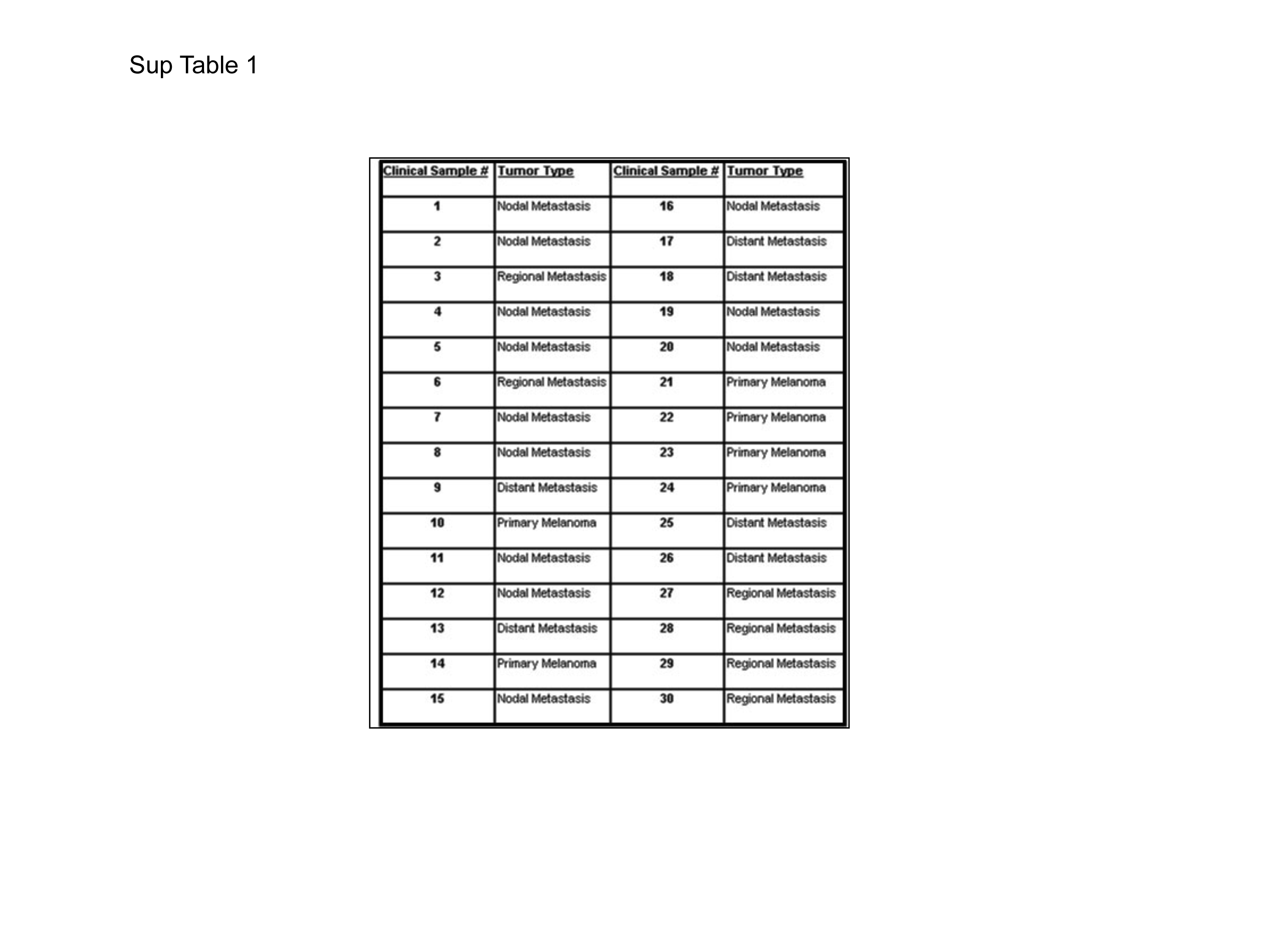

Supplement: Table S1 — Description of human clinical samples. (0.72 MB TIF) [file pone.0013779.s004.tif]

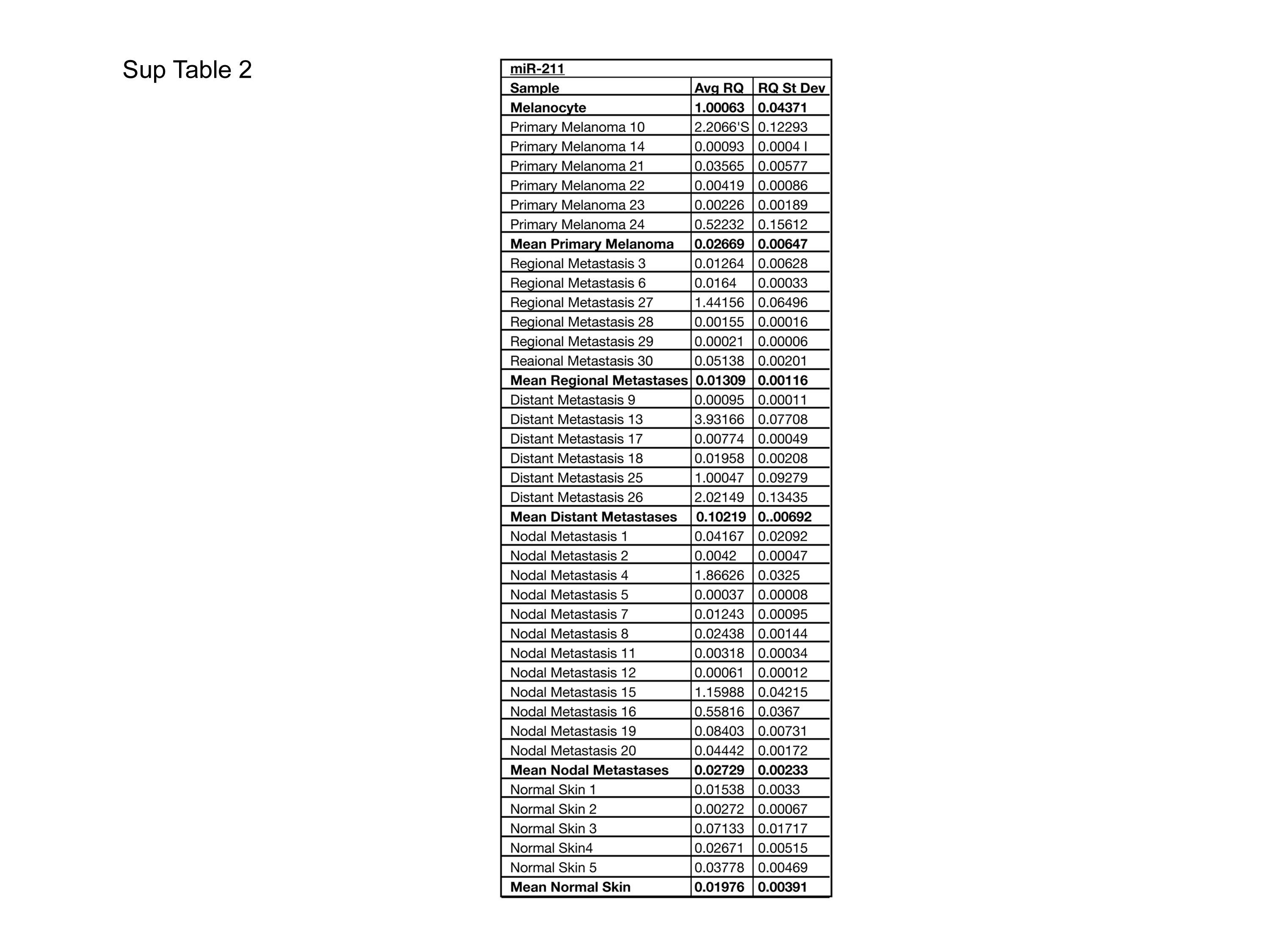

Supplement: Table S2 — miR-211 expression levels in clinical samples. Note: Two-tailed t-test comparisons for miR-211 by mean relative quantification levels of melanocyte and primary melanoma, as well as regional, distant, and nodal metastatic melanoma were all statistically significant at P<0.000001. (0.84 MB TIF) [file pone.0013779.s005.tif]
